# Platelet Storage—Problems, Improvements, and New Perspectives

**DOI:** 10.3390/ijms25147779

**Published:** 2024-07-16

**Authors:** Natalia Trochanowska-Pauk, Tomasz Walski, Raghvendra Bohara, Julia Mikolas, Krystian Kubica

**Affiliations:** 1 Department of Physics and Biophysics, The Faculty of Biotechnology and Food Science, Wrocław University of Environmental and Life Sciences, 50-375 Wrocław, Poland; natalia.trochanowska-pauk@upwr.edu.pl; 2 Department of Biomedical Engineering, Faculty of Fundamental Problems of Technology, Wrocław University of Science and Technology, 50-370 Wrocław, Poland; krystian.kubica@pwr.edu.pl; 3 Centre for Interdisciplinary Research, D.Y. Patil Educational Society, Kolhapur 416006, India; raghvendrabohara@universityofgalway.ie; 4 Faculty of Medical Sciences in Zabrze, Medical University of Silesia in Katowice, 41-800 Zabrze, Poland

**Keywords:** platelet storage lesion, shelf-life, platelet storage, blood banking, platelet concentrates

## Abstract

Platelet transfusions are routine procedures in clinical treatment aimed at preventing bleeding in critically ill patients, including those with cancer, undergoing surgery, or experiencing trauma. However, platelets are susceptible blood cells that require specific storage conditions. The availability of platelet concentrates is limited to five days due to various factors, including the risk of bacterial contamination and the occurrence of physical and functional changes known as platelet storage lesions. In this article, the problems related to platelet storage lesions are categorized into four groups depending on research areas: storage conditions, additive solutions, new testing methods for platelets (proteomic and metabolomic analysis), and extensive data modeling of platelet production (mathematical modeling, statistical analysis, and artificial intelligence). This article provides extensive information on the challenges, potential improvements, and novel perspectives regarding platelet storage.

## 1. Introduction

Platelet concentrate (PC) transfusion is often used to treat platelet (PLT) function disorders and thrombocytopenia. Patients may receive PLT transfusions to enhance hemostasis during spontaneous, traumatic, or perioperative bleeding [1]. Hemostasis and thrombosis are not the only two processes in which PLTs play an essential role, but they perform many other functions, including promoting the inflammatory and immune response, recruiting leucocytes and progenitor cells to sites of vascular injury and thrombosis, storing, producing, and releasing pro-inflammatory, anti-inflammatory, and angiogenic factors as well as microparticles into the circulation [2,3].

**Figure 1:**
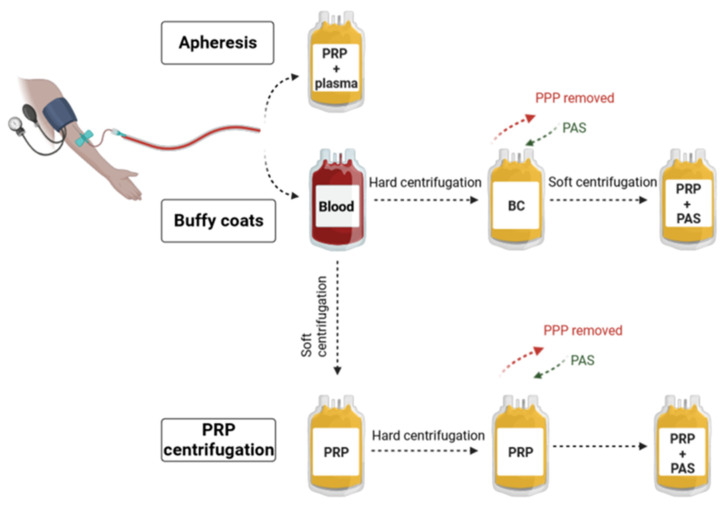
Platelets (PLTs) isolation methods for transfusion purposes. According to standard procedures, PLTs are obtained from volunteer donors and can be collected in three ways. The first method is apheresis, which is the process of donation using a programmable machine called a cell separator (connected to the donor) to collect PLTs directly from the bloodstream. The second is the isolation of PLTs from buffy coats (BCs). The third and last is based on the centrifugation of platelet-rich plasma (PRP). The difference between the PRP and BC methods lies in the centrifugation protocol. In the BC method, whole blood is hardly centrifuged, which results in the separation of three fractions: red blood cells, BC, and platelet-poor plasma (PPP). The PPP is removed, leaving 50–70 mL of plasma [18]. Then, BC is pooled (or not if processed as a single unit) and transferred to another bag, followed by resuspension in platelet additive solution (PAS) (or plasma). Finally, soft spinning of BC is performed to discard the remaining sedimented red and white blood cells, while PRP is transferred to the final bag of PLT concentrate. In turn, using the PRP method requires whole-blood soft centrifugation. The PRP is transferred to another storage bag, and hard centrifugation is performed, followed by PPP removal and PLTs resuspension in PAS (or plasma) [19]. Created with BioRender.com.

According to standard procedures, PCs are obtained from volunteer donors and can be collected from whole blood with the platelet-rich plasma (PRP) method or the buffy coat (BC) method. Additionally, PLTs can be obtained by plateletpheresis, harvesting the PLT but returning all other blood cells to the donor [4] (Figure 1). Each of the described procedures has some limitations and disadvantages [5,6,7,8] (Table 1). Experimental models have shown that the procedure of PLTs isolation affects their activation and vital functions that are strictly combined with storage. It is proved that better-quality PCs, measured as the level of PLTs activation, can be obtained by both BC and the apheresis methods [7]. 

After 7 days of storage, PLTs show a reduction in their therapeutic efficacy. It is related to morphological, biochemical, and functional changes observed as, for example, the loss of discoid shape, decreased mean platelet volume, increased volume and density heterogeneity, the increased release of PLT granules and cytosolic proteins, increased procoagulant activity, and altered glycoprotein (GP) expression. All of the effects mentioned above are typical for the development of platelet storage lesions (PSLs) [8,9,10], which have been well-reviewed before [11,12,13]. PSL progresses from blood collection to transfusion, and it is also accompanied by increased oxidative stress and platelet microparticles (PMPs) formation, complement activation [14], a decrease in glucose and ATP, higher levels of lactate hydrate [12,15,16], and irreversible PLT activation [17], which, lastly, limits the availability and safety of the PC.

Over recent years, PRP has gained significant attention and application in the field of regenerative medicine. Studies have demonstrated that PRP is rich in PLT-derived growth factors and maintains normal levels of plasma fibrinogen, both of which work together to enhance the regenerative process. Additionally, the cost-effectiveness of PRP compared to traditional therapies makes it a favorable option for many healthcare providers [20]. Nevertheless, this work does not address this topic. This review specifically focuses on the methodologies for storing PLTs for transfusion purposes, ensuring the availability of this critical component for emergency and life-saving interventions.

Literature reports indicate that the age of donors also affects the storage duration and quality of PCs. Population aging is a global phenomenon with different impacts in developed and developing countries. According to data published in 2021, stored PLTs from older male donors exhibit increased Krebs cycle metabolism, indicating metabolic storage lesions. These PLTs exhibit similar post-transfusion recoveries at 24 h but have a shorter survival time in the bloodstream of autologous recipients [21].

**Table 1:** Limitations of current methods of PLTs isolation and potential recommendations for enhancement.

PLT Collection Method	Challenges	Recommendations
Apheresis	- High cost [22,23]. - Time-consuming [22,24]. - Requiring double puncture of the patient. - One patient = one unit [22].	- Reducing the expenses associated with utilizable materials. - Triple apheresis production of three therapeutic units during one collection (in vivo studies required) [25].
Buffy coats and conventional methods	- Pooling (~5 donors) [22]. - Post-storage leukoreduction. - The need to use PAS [26,27,28]. - Strong PLT activation [8,16,17]. - Risk of bacterial contamination [22].	- Minimizing the number of donors. - Performing the leukoreduction after or during blood collection. - Optimization of the centrifugation speed. - To reduce the level of PLT activation. - Modification of PAS composition for enhanced resemblance to natural plasma. - Further studies on pathogen reduction technology.

The World Health Organization (WHO) highlights the issue of blood wastage in its report titled ‘Towards Self-Sufficiency in Safe Blood and Blood Products based on Voluntary on-Remunerated Donation’. In 2013, WHO data from 148 countries showed that 5.2% of blood and blood products were utilized [29]. However, the 2022 WHO report estimates that approximately 23% of blood and blood product supplies worldwide were discarded solely due to exceeding their expiration date (Global Status Report on Blood Safety and Availability, WHO, 2022). This global average masks significant regional variations. For instance, Malaysia recorded a discard rate of around 6% for PCs in 2012 [30], whereas India reported a much higher rate of 37% [31]. In the USA, approximately 10% of produced PCs are discarded [32], while in Iran in 2015, this percentage was only 3.8% [33]. Efforts to enhance PLT banking are multifaceted due to the complex nature of the PLT storage issue. Research primarily aims to extend the storage duration of PLTs, enhance their quality, and minimize the wastage of valuable biological material. Therefore, exploring avenues to prolong the lifespan of PLTs is imperative.

### Solutions Can Be Found by Carrying out Research on Aspects of the 4Es

Currently, research in the field of PLT storage can be divided into several main groups. Researchers are encouraged to conduct studies on any of the following 4E approaches:

1. Extending storage conditions: This involves investigating various factors that can impact storage conditions, including exploring optimal storage temperatures and evaluating the effectiveness of using gas-permeable bags.
2. Enabling additive solutions: This includes designing synthetic compositions that can effectively suspend and store PLTs, aiming to create improved additive solutions for long-term storage.
3. This involves exploring alternative methods for blood PLT storage and testing, including iPSC, photobiomodulation, -omics, and microRNA (miRNA) technology. These investigations hold promise for discovering new insights and improving existing storage methods.
4. Employing statistical modeling and big data technologies: Utilizing these methods can result in a more efficient and effective blood management system. These methods offer the potential for valuable insights and better decision-making processes.

By actively pursuing research within these 4E approaches, significant advancements in PLT storage can be made. These efforts have the potential to extend the storage time of PLTs, enhance the quality of stored PLTs, and ultimately reduce the wastage of this critical biological resource. Moreover, the exploration of these avenues may lead to the development of novel storage methods and innovative solutions, ultimately improving the overall management of blood products.

This narrative review provides a summary of strategies related to PSL and problems, improvements, and new perspectives for transfusion medicine. We searched PubMed in May 2024 using the primary search phrase ‘(platelet storage lesion) AND (platelet concentrates) AND (platelet OR platelets)’. We also performed searches with additional key terms (for example, ‘pathogen reduction’ and ‘pathogen inactivation’, ‘aging’, ‘cold storage’, ‘Platelet additive solutions (PASs)’, and ‘cryopreservation’) to identify articles specifically relevant to each section of this review. This initial search returned 1375 results. Only peer-reviewed articles in English were considered. We checked the relevance of the titles/abstracts of the retrieved articles, identified by manually searching reference lists. Where multiple articles reported similar findings, priority was given to those most recently published. In total, 143 articles were deemed by the authors as most relevant to PSL—problems, improvements, and new perspectives and were included in this narrative review.

## 2. Platelets: Small Cells with Great Importance in Health and Disease

PLTs are the smallest blood cells produced in the bone marrow from stem cells called megakaryocytes (MKs). They have a diameter of 2–3 μm, an average volume of about 7 μm^3^, and a thickness of 0.5 μm. The normal count of PLTs in human blood is between 150 and 400 × 10^9^ per liter. Two-thirds of the PLTs are in the blood, while the remaining one-third is stored in the spleen. An adult can produce around 10^11^ PLTs daily. PLTs are the second most numerous cells in the blood and play a crucial role in responding to vascular injuries. They also signal white blood cells to initiate the inflammatory process. The lifespan of a PLT in the bloodstream is 5 to 9 days. Younger PLTs are larger than older ones and are removed by macrophages in the spleen and liver (Kupffer cells) [34,35].

The primary and well-known function of PLTs is their participation in the process of coagulation and hemostasis [35,36]. Hemostasis involves several processes aimed at keeping blood in a fluid state within the vascular system. When there is damage to a blood vessel, these processes prevent the blood from leaking out by forming a clot—initially, a PLT plug (primary hemostasis), followed by a fibrin clot (secondary plasma hemostasis). In the body, these events occur nearly simultaneously and are closely linked [35,36].

The most important stage of hemostasis is clot formation. It involves the enzymatic conversion of fibrinogen (Factor I) into fibrin (Factor Ia), facilitated by thrombin. Thrombin circulates in the blood in an inactive form as prothrombin (Factor II) and can be activated via two pathways: intrinsic or extrinsic. The extrinsic pathway is initiated by tissue factor (TF), released into the blood by damaged tissue cells. The intrinsic pathway begins when PLTs come into contact with negatively charged surfaces (e.g., exposed collagen), leading to the activation of Factor XIIa [37].

Excessive activation of PLTs has been observed in many diseases, such as atherosclerosis, cerebral ischemic events, and acute coronary syndromes, leading to thrombosis within the vessel lumen. This excessive activation affects the progression of these diseases. The routine use of antiplatelet drugs has significantly improved patient prognosis. Current research is focused on identifying both known and new molecules that can affect previously understood as well as entirely new pathways of PLT aggregation [38].

PLTs have the ability to directly bind pathogens by expressing receptors that recognize pathogen-associated molecular patterns (PAMPs) [39,40]. They can eliminate pathogens through encapsulation and antimicrobial peptides [41,42]. PLTs are often called “circulating guards” in the literature because they interact with immune complexes and form leukocyte–PLT aggregates that immobilize pathogens [43]. Many studies show a connection between a decrease in PLT count (thrombocytopenia) and infections [41,44]. Severe thrombocytopenia during sepsis is usually a poor prognostic indicator, suggesting that PLTs play a crucial role in critically ill patients [44]. The mechanisms of thrombocytopenia during sepsis are still being discussed, with excessive PLT destruction, sequestration, or bone marrow production defects being proposed as causes [41].

When tissues become infected, inflammation can result from the direct or indirect harmful effects of certain microbial products. PLTs are the first cells to gather at the site of vascular injury, setting off inflammatory processes by releasing substances such as histamine, serotonin, pro-inflammatory chemokines, and cytokines. They also play a role in the later stages of inflammation. Through the expression of various functional immune receptors, PLTs influence the immune response, contributing to innate immunity [45]. These receptors allow PLTs to interact with immune cells in the vascular endothelium and spleen [46].

When the body experiences inflammation and oxidative stress, tiny cell particles called microparticles (MPs) are produced. These MPs are released by all cells in the blood. During inflammatory processes, activated PLTs release PMPs, which vary in size from 0.2 to 1 μm. High levels of PMPs in the blood are linked to many diseases [47]. PMPs often attach to granulocytes and lymphocytes, causing these cells to increase the expression of adhesion molecules and their ability to engulf foreign particles. PMPs also trigger the secretion of cytokines and influence the growth of new blood vessels [47,48].

PLTs can interact with pathogens by expressing antimicrobial molecules. This helps them to kill pathogens, and they can also engulf and store them, facilitating interaction with immune cells [49,50]. The ability of PLTs to produce several antimicrobial molecules emphasizes their vital role in the initial response to detected abnormalities. These small blood cells act as a bridge between innate and adaptive immunity.

PLTs have long been believed to play a role in transfusion-related immunomodulation (TRIM), independent of leukocytes [51,52]. In a mouse model of immune thrombocytopenia, PLT transfusion was found to help stabilize PLT counts [53]. In other studies, Ki et al. (2018) showed that stored PLT concentrates affected myeloid dendritic cells in various infection models. PLT transfusion led to the different regulation of co-stimulatory molecules and cytokine production depending on the specific infection model [54]. Although PLT transfusions are essential and beneficial, it is important to be aware of their potential to modulate the immune system in different ways, as this can also lead to adverse events [55,56].

## 3. Storage Conditions for Platelet Concentrates

Storage conditions play a key role in maintaining PLT functionality. In contrast to red blood cells, storage at low temperatures compromises the viability of stored PLTs as it alters the ability to shapeshift and activate under various conditions [57]. The quality of the PC and its in vivo effectiveness are also strongly dependent on the type of storage medium used or the application of the pathogen inactivation (PI) system [58].

### 3.1. Standard Storage Conditions

The guidelines for PLT storage recommend a temperature between 20 and 24 °C. These limits came from a study performed 60 years ago, in which the effect of storage at different temperatures for different durations on the in vivo recovery and survival of PLTs was demonstrated [58]. The standard temperature for PLT storage in blood banks is 22 ± 2 °C with constant gentle horizontal agitation. This procedure allows storing PCs for up to 5 days in a closed system. This time could be extended to 7 days if appropriate packs and PC combinations were used. Due to concerns of bacterial contamination, the use of 7-day PCs requires an assay to exclude bacterial contamination prior to transfusion or application of a pathogen inactivation procedure [59,60]. If any manufacturing step includes PLTs collecting in an open system, the PC should be used immediately after collection. Sometimes, storage is unavoidable. The PC should then be stored at a standard temperature of 22 ± 2 °C with continuous agitation and used within 6 h [61].

During the storage procedure, the PLTs should be gently agitated. If some interruptions appear (e.g., the integrity of the hermetic seal is broken) due to transportation, equipment failure, processing, etc., PCs are still suitable for use; therefore, the total time of interruption cannot be longer than 24 h, and a single interruption should not last longer than 8 h. If the conditions are fulfilled, the components are suitable for use with an unchanged shelf life [61]. According to the most recent studies, constant agitation is still unavoidable. The shear forces induced by agitation keep the GPVI receptor down-regulated, attenuating PLT-spreading capacities during storage [62].

The effects of PLT storage at low temperatures (less than 20 °C) were demonstrated in two key papers. Gottschall and Aster (1986) [63] stored PLTs at 18 °C, 19.5 °C, and 21 °C for 72 h. They found a significant effect on the PLTs’ mean life span, which was reduced from 8.1 days at 21 °C to 5.2 and 1.9 days after storage at 19.5 °C and 18 °C, respectively. These results clearly showed the need for quality control of temperature in PLT storage. Even if in vitro markers of PLT quality indicate a positive impact of temperatures below 20 °C, the most important is post-transfusion survival in vivo. In another study, PLTs were stored for 5 days at room temperature with or without 17 h of exposure to 16 °C or 12 °C. Post-transfusion recovery was slightly reduced after exposure to 16 °C or 12 °C (from 49% in the control group to 43% and 38%, respectively) [64]. However, according to Gottschall’s research, PLT post-transfusion survival was also reduced from 6.5 to 3.5 and 2.0 days after exposure to 16 °C or 12 °C, respectively [65,66].

Table 2 outlines the drawbacks associated with the current approaches to storing PLTs under standard conditions, along with proposed strategies for enhancing this storage process.

### 3.2. Cold Storage

The effects of PLT storage at low temperatures were demonstrated in many articles [65,73,74]. Currently, the temperature studied most often for cold storage is 4 °C, with or without agitation. One of the essential advantages of refrigerated storage of PLTs is that it reduces the risk of bacterial growth and provides a way to decrease the number of transfusion-transmitted infections [57]. On the other hand, the main problem of cold-storage (under 15 °C) is reduced PLTs’ in vivo performance [75,76]. After transfusion, they are almost immediately cleared from the bloodstream. This is initiated by changes in GPIbα, the receptor for the von Willebrand factor. The GPIbα activation induces apoptosis, initiates hemostatic responses upon rewarming, and promotes clearance of PLTs from the circulation, which is the reason why cold storage is excluded from routine procedures of transfusion medicine [77]. The molecular mechanism of this process is already well known. In 2012, Gitz et al. examined the change in GPIbα distribution using Förster resonance energy transfer by time-gated fluorescence lifetime imaging microscopy. Their results showed that cold storage induced deglycosylation of the GPIbα ectodomain, exposing N-acetyl-D-glucosamine residues sequestered with GM1 gangliosides in lipid rafts. The study aimed not only to show the molecular mechanism of the deglycosylation of GPIbα but also to find a solution to protect PLTs from this process. It was concluded that inhibition of GPIbα clustering by the depletion of N-acetyl-2,3-dihydro-2-deoxyneuraminic acid and arachidonic acid provides a simple means to prevent damage that compromises the recovery and survival of cold-stored PLTs [78]. Recently, Marini et al. published the results of in vitro and in vivo experiments, examining the impact of apoptosis inhibition on the hemostatic functions and survival of cold-stored PLTs. They observed that blocking the apoptotic signal transduction induced by GPIbα clustering or the activation of caspase 9 did not negatively affect the functionality of cold-stored PLT. Indeed, inhibiting the apoptotic signal mediated by GPIbα clustering with a RhoA inhibitor better preserved the release of δ granules, PLT aggregation, adhesion, and the ability to form stable clots compared to the control group. They also noted a significant improvement in the half-life of refrigerated PLTs upon inhibition of the intracellular signal induced by GPIbα clustering. The presented results provide hope that combining cold storage with apoptosis inhibition could be a promising strategy to extend the storage time of refrigerated PLTs without compromising their hemostatic functions or survival [79]. Cold-stored PLTs undergo a process similar to agonist-induced activation; however, the similarity between these two events seems to be restricted to the final stages of the signaling cascade, making our understanding of cold activation intriguing and challenging. Cold storage can disrupt calcium ion pumps and channels in the PLT membrane, resulting in changes to intracellular calcium levels. This altered calcium homeostasis has the potential to impair PLT activation pathways, thereby affecting downstream signaling cascades essential for proper PLT function.

**Figure 2:**
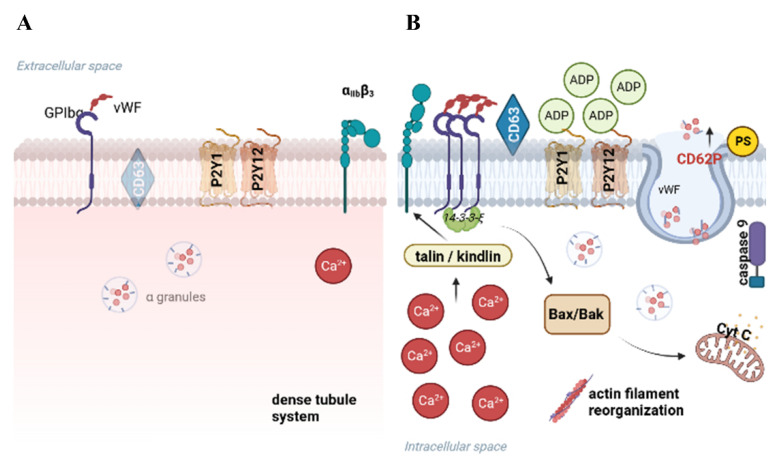
The influence of thermal conditions on PLTs during storage. When PLTs are stored at room temperature (**A**), they typically retain their discoid shape and display diverse surface receptors. During cold storage, the deceleration in the metabolic rate, accompanied by changes in the cytoplasmic membrane and modifications in the expression of surface receptors, is observed (**B**). Created with BioRender.com.

Furthermore, PLT cytoskeletal elements, particularly microtubules and microfilaments, play a crucial role in various PLT functions, such as shape change, granule release, and aggregation. The reduced temperature during cold storage may lead to disorganization of the actin cytoskeleton, which can impact both PLT structure and function. Additionally, cold storage influences PLT-PLT interactions as well as the formation of PLT aggregates. Altered membrane properties, receptor expression (inter-alia CD62P, CD63, GPIbα, GPαIIβ3), and changes in the cytoskeleton can modulate the strength and stability of PLT aggregates, which are pivotal for effective clot formation. Low temperatures also trigger the activation of PLT through processes like the release of PLT granule contents and the presentation of phosphatidylserine. Moreover, cold temperatures exert considerable influence on the metabolic processes of stored PLTs. Overall, understanding the physiological consequences of cold storage on PLTs is essential for optimizing storage conditions and preserving their functionality, ensuring their efficacy in transfusion therapies and hemostatic processes [80] (Figure 2, Table 3). Despite these limitations and concerns, the FDA recently issued guidance that includes a notice of exceptions and alternatives for conventional PC storage methods. This guidance permits the storage of apheresis PLTs at 1 to 6 °C for up to 14 days from the date of collection as an alternative procedure for the treatment of active bleeding in cases where conventional PLTs are unavailable, or their use is impractical [81]. A potential application of cold-stored PLTs may involve their use during cardiac surgeries. In such cases, the prolonged survival of PLTs may not be as crucial as their functionality during the procedure and up to 24 h afterward when hemodynamic balance is disrupted [82].

### 3.3. Cryopreservation

In this method, PLTs, after supernatant removal, are stored at −80 °C with the addition of dimethyl sulfoxide (DMSO). According to the procedures, cryopreserved PLTs (CPP) can be stored for up to 2 years. PLTs are rapidly transfused after thawing and resuspension in an adequate saline solution [85]. CPPs show hemostatic functionality, and their clinical short-term efficiency has been proven [83,86,87], but they do not meet the FDA survival criteria for 24 h. In fact, cryopreservation induces undesirable processes in PLT, such as increased activation, unresponsiveness to agonist stimulation, and decreased levels of GPIbα, GPVI, and integrin αIIbβ [88,89]. Furthermore, in 2017, Eker et al. observed strong PMP formation during cryopreservation, while there was no difference in PLT numbers before and after the procedure. Significantly higher and earlier thrombin formation occurred in the samples. At the same time, the viability of PLTs was reduced [90]. Similar effects, such as the formation of microparticles and weakened aggregative capacity, were observed by Gavioli et al. Furthermore, it was noted that within the first 3 h after thawing, irreversible changes occur in the biomolecular structure of CPP [91] (Table 4).

However, there are clinical trials that aim to implement PLT cryopreservation technology, for example, by the Australian and New Zealand Intensive Care Research Centre [92,93]. In this phase III multicenter, blinded, randomized controlled clinical non-inferiority trial, it is investigated whether CPP will be at least as effective and safe as conventional liquid-stored PLTs in treating active bleeding due to surgery. The positive outcome of these studies will result in a reduction in overall PC wastage, allow smaller hospitals to provide PLT transfusions, and improve the cost-efficiency of both storage and surgical procedures.

The effects of storage conditions on therapeutic PLT products at room temperature (5–7 days), cold (5–7 days), or frozen (months-years) when compared with PLTs present in fresh PRP were reviewed by Hegde et al. (2018). Their studies indicated that, overall, room temperature storage preserves more PLT characteristics, whereas cold and DMSO frozen storage leads to various functional and structural impairments. While room temperature storage maintains normal PLT size and volume, it does reduce pH and delay clot formation and thromboxane A2 production, although swirling ability is preserved. In contrast, cold storage decreases PLT count and volume, mildly reduces pH, and increases microparticle (MP) content and granule secretion while improving aggregation response. Cryopreservation also decreases PLT count and volume, maintains a mildly reduced pH, and significantly increases MP content, causing cytoskeletal damage. Across all storage conditions, phosphatidylserine externalization, membrane CD40L, and Factor V binding increase. ATP production efficiency is poor in room temperature storage and modestly reduced in both cold and cryopreserved storage [94]. However, current clinical research indicates that cold-stored PLTs have the worst survival and recovery rates after 2 and 7 days of storage, with significantly better outcomes for CPP and room temperature-stored PLTs, with a slight preference towards the latter [94,95,96,97]. These findings emphasize the need for careful consideration of storage conditions to optimize the therapeutic potential of PLT products.

### 3.4. Lyophilized Platelets

Lyophilized platelets (LPs) have undergone lyophilization, a process involving freezing and drying under reduced pressure. This converts the cells into a dry powder, allowing for their long-term storage at room temperature with a shelf life of up to 24 months. Over the years, researchers have refined and modified the lyophilization process to develop the most effective procedures. PLTs were most commonly fixed with paraformaldehyde and then lyophilized in a solution of serum albumin [98,99]. Protocols involving lyophilization with or without a cryoprotectant have been reported [98,100].

The efficacy of LPs in PLT adhesion and bleeding reduction in thrombocytopenic models has been evaluated in numerous studies [98,99,100,101]. Preliminary research has led to clinical trials assessing the safety of LPs in healthy patients (NCT02223117, phase I) [102]. This clinical trial, which has been completed, was a randomized, blinded, dose-escalation safety trial of autologous thrombosomes, and no serious adverse events were reported. The latest ongoing trials aim to evaluate the efficacy of LPs during cardiopulmonary bypass surgery [103].

The evaluation of LPs has also been conducted using animal models. In a study by Gogg et al., the effects of CPP and LPs were compared in a canine model [104]. Most of the dogs had primary or secondary immune thrombocytopenia. The study indicates that LPs are comparable to CPP in managing bleeding in thrombocytopenic dogs. Within the acute period (1 h post-transfusion), LPs might be more effective than CPP in reducing bleeding scores and preventing a drop in hematocrit, although the clinical significance of these minor differences remains unclear. Future research should aim to compare LPs with fresh PC or fresh whole blood, assess LPs’ effectiveness in scenarios such as trauma and intraoperative bleeding, and explore the potential benefits of combining LPs with other hemostatic treatments, including antifibrinolytic agents [104].

To enable the use of LPs beyond thrombocytopenic patients, it was necessary to investigate the interactions between LPs and normal PLTs. In 2022, Schnoor et al. published a study characterizing LPs in comparison to unaltered PLTs [105]. The study analyzed the activity of key PLT receptors, phosphatidylserine exposure on the PLT surface, and fibrinogen binding capacity. Additionally, the interaction between LPs and PLTs was assessed ex vivo through aggregation and adhesion experiments. These experiments shed new light on the mechanisms of LP and PLT interactions. The analysis indicated that LPs retained the receptors responsible for adhesion. A significant portion of LPs exhibited increased phosphatidylserine exposure and fibrinogen binding. The evidence clearly demonstrates that these LPs interact with untreated PLTs and significantly impact their function. Interestingly, the interactions have opposite effects on aggregation versus adhesion. LPs have a robust anti-PLT effect in terms of aggregation, leading to significant decreases in PLT aggregation when LPs are mixed with untreated PLTs. This trend was consistent across different PLT counts, indicating that LPs inhibit the aggregation function of untreated PLTs [105].

### 3.5. Platelet Additive Solutions (PASs)

The additive solutions are electrolyte solutions with different types of specific ingredients that could modify PLT quality. The popularity of PASs has continuously increased in recent years. The idea of using PASs was to store as small an amount of plasma with PLTs as possible. Therefore, PAS is used to replace plasma as a storage medium for PLTs. Due to the use of the procedure, some benefits are observed. First, a reduction in the amount of plasma transfused with PLTs is possible, which means a lower risk of allergic transfusion reactions with equivalent clinical efficacy to control bleeding [22,26]. Another advantage is that photochemical treatment for the inactivation of bacteria and other pathogens can be applied, and as a result, it improves storage conditions. PASs are used for all types of stored PLTs (obtained by both apheresis and BC method). Nowadays, the storage medium is composed of 20–50% plasma and 50–80% PASs [106].

There are several types of PAS. The simplest is PAS-II. It contains only sodium chloride, sodium citrate, and sodium acetate. PAS-II with phosphate as a buffer is named PAS-III. Due to phosphate in the solution, the metabolism of glucose increases, leading to higher lactate production [27]. To reduce the adverse effects of PAS-III potassium and magnesium were added and named PAS-IIIM, which is an effective substitute for plasma [28]. Additionally, Aurich et al. 2022 demonstrated that cold-induced cytoskeletal disorganization can be prevented by storage media supplementation with at least 10 mM magnesium ions (Mg^2+^) [84].

New solutions for PAS components are constantly sought. One of the recent discoveries is the positive effect of resveratrol on stored PLTs. The addition of this natural ingredient at a concentration of 10 μM contributed to the reduction of PLT activation and diminished free mtDNA during 7 days of storage [107], which is a direct marker of oxidative stress [108]. Hence, resveratrol could serve as a potential additive to enhance the preservation quality of stored PCs.

Multiple studies have shown a positive impact of different PAS on PLTs in vitro quality, but only a few authors investigated the parameters of PAS-treated PLTs after transfusion (in vivo) [77]. This type of research is clinically essential and should be studied in great depth (Table 5).

### 3.6. Pathogen Reduction Technologies—Is It a Blessing?

Currently, three pathogen inactivation systems are commercially available to produce pathogen-reduced PCs. All of them use UV light in the presence or absence of a photosensitizer. These technologies were widely reviewed in the literature [67,109,110], which is why only key issues necessary for the context of this review are provided.

INTERCEPT™ Blood System (Cerus Corporation, Concord, CA, USA) is a PI system that combines the use of amotosalen (a psolaren) and UVA light. Amotosalen can penetrate cellular and nuclear membranes. It can develop a noncovalent link between pyrimidine bases in DNA and RNA chains. A photochemical reaction is induced by exposition to UVA light (320–400 nm) and causes the transformation of the preexisting link into an irreversible covalent bond, preventing DNA replication and RNA transcription. The results of in vitro and in vivo studies have not demonstrated any toxicologically relevant effects of PCs prepared by the INTERCEPT™ system [68,111]. However, some publications reported that upon INTERCEPT™ treatment, a reduction in PLT function is observed [112,113,114,115]. INTERCEPT™ has been shown to deregulate the expression of anti-apoptotic genes, modulate the PLT characteristics and aggregation response to physiological agonists, and the level of activation [113]. Moreover, INTERCEPT™ can modulate the PLT mRNA transcriptome [116].

Another PI system is MIRASOL^®^ (Terumo BCT, Lakewood, CO, USA). In this method, riboflavin is a photosensitizer for generating reactive oxygen species (ROS) upon UVA/UVB (270–360 nm) irradiation, which damages guanidine nucleotide bases. Riboflavin is considered safe and does not need to be removed after illumination [69,117,118].

The third commercially available PI system is THERAFLEX (MacoPharma, Tourcoing, France), which uses a method based on UVC light in combination with strong agitation. Strong agitation facilitates light penetration, and the whole process does not require a photosensitizer. UVC radiation acts directly on nucleic acids by inducing pyrimidine dimers and blocking DNA replication [70,119].

Pathogen reduction technology demonstrates high effectiveness in the inactivation of most tested pathogens, including West Nile virus [120], Zika virus [121], dengue virus [122], and hepatitis-E virus [123]. INTERCEPT™ demonstrates a higher inactivation capacity for the bovine viral diarrhea virus and pseudorabies virus than MIRASOL^®^. Both technologies show similar log reductions for hepatitis-A virus and porcine parvovirus. Due to the applied chemistry, PI systems can inactivate pathogens that contain nucleic acids. As a consequence, they are ineffective for prions and transmission of variant Creutzfeldt–Jakob disease [124].

The other side of PI systems also warrants consideration, as they have not undergone sufficient testing. Moreover, the complete molecular mechanism behind these systems remains unclear. It is already established that PI treatment can result in various forms of damage to PLTs, ranging from changes in membrane integrity and signaling pathways to impaired functionality of miRNAs. This damage has the potential to diminish the recovery and survival rates in healthy patients [32,125] (Table 6). Currently, several clinical trials utilizing PI systems are underway, focusing on whole blood and red blood cells [71,126,127].

Based on the research conducted thus far, it is evident that PI-treated PLTs exhibit notable differences compared to untreated ones. To comprehensively understand the functional alterations in PLTs, it is imperative to conduct clinical trials involving the transfusion and further in vivo monitoring of cells treated with pathogen reduction technology. Hopes for a thorough explanation of the molecular mechanism of PI systems give proteomic analyses and metabolomics [18].

Considering solely the inventory management aspects of blood products, PRT can contribute to waste reduction. This was demonstrated, among others, in the study by Fachini et al. 2021. The authors proved that only through the implementation of PI systems at Sírio-Libanês Hospital in São Paulo, Brazil, the discard rates of blood PCs decreased from 6% to 3%. A further decline, to 1.2%, was noted after extending the storage time to 7 days. A significant decrease in adverse transfusion events associated with the implementation of PRT was observed [128].

**Table 6:** Limitations of pathogen reduction technologies and potential recommendations for enhancement.

Challenges of PRT	State of Art	Recommendations
- Only commercially available. - UV light is dose-dependent and causes PLT activation and aggregation [70,112]. - Molecular mechanism of action is not fully understood [113,116,129]. - Causes alterations in membrane integrity [113,116,129]. - Induces miRNA changes [113,116,129].	- Lower bacterial contamination [67,68,69,109,111,112]. - Higher quality of PRT concentrates [115]. - Molecular changes during PRT use [113,116,129].	- Reducing costs to broaden access for a larger number of blood centers. - Exploring diverse irradiation approaches to protect against bacterial contamination. - Research for the explanation of the molecular mechanism of action (proteomic and metabolomic analysis).

## 4. Novel Strategies for Platelet Storage Lesions and Research

### 4.1. Induced Pluripotent Stem Cells

COVID-19 pandemic restrictions, like lockdowns, reduced societal mobility, and sanitary and epidemiological requirements have slowed and reduced the number of donations, which has influenced a shortage of blood products. The pandemic has drawn significantly greater attention to the ex vivo production of transfusable blood cells. Beyond the pandemic, long weekends, holidays, and vacations also contribute to a decrease in blood donations. Concerns about PLT product supplies had already arisen due to their short shelf life (up to 7 days), supply–demand imbalance in aging societies of developed countries, and alloimmune-mediated PLT transfusion refractoriness. Consequently, there are high expectations for ex vivo products to address these issues. One approach involves using artificial components, such as liposomes, to create particles that mimic the function of PLTs. However, this solution has so far been ineffective in vivo, as the body still recognizes the transfused preparations as foreign, leading to increased aggregation, which is hazardous for patients. Another promising approach under investigation is the differentiation of PLTs from stem cells, particularly induced pluripotent stem cells (iPSCs) in the laboratory. Human iPSCs were first used in 2006 [130]. Since then, interest in using these cells to produce PLTs has steadily increased due to their self-renewal capacity and pluripotency. They hold significant potential for use in transfusion medicine and other therapies. One of the key advantages of iPSC-derived PLTs is their potential for large-scale production, providing a sustainable and scalable source of PLTs for patients in need of transfusion. These cells enable the delivery of patient-specific products or adjustments to desired phenotypic characteristics.

One of the major challenges in the potential clinical use of iPSCs is the establishment of an appropriate process for generating blood cells, including PLTs. This is a significant challenge because approximately 10^11^ cells are transfused to a patient during a single transfusion. Without gene transduction, MKs practically do not proliferate. Therefore, the development of a cell source capable of expansion is crucial for PLT production. Feng et al., in 2014, achieved the generation of universal PLTs from iPSCs with a deletion of the β2-microglobulin gene and demonstrated that a single MK precursor generates approximately six PLTs [131]. Moreau et al. achieved chemically defined large-scale production of MKs from iPSCs, where a single MK released approximately five PLTs [132]. Currently, a single cell from the human adipose-derived mesenchymal stromal/stem cell line (ASCL) generates from 5 to 10 ASCL-PLTs [133]. ASCL-PLTs have similar characteristics to peripheral PLTs and may additionally serve as mesenchymal-like cells. Tozawa et al. investigated the in vivo dynamics of ASCL-PLTs and standard PCs by administering these cells to irradiated immunosuppressed NSG mice (2.0 Gy, for 7 days). Blood samples from transfused mice were analyzed before the procedure and at 30 min, 2, 4, 6, and 24 h post-transfusion. The kinetics of ASCL-PLTs mirrored that of standard PCs. According to the study, no spontaneous PLT aggregation before agonist-induced stimulation was observed; there is a possibility that ASCL-PLTs may lead to thrombotic complications. The published protocol represents a straightforward technique, thereby bolstering the clinical implementation of the methodology. Thus, it is important to conduct further studies to optimize ASCL-PLTs as candidates for clinical applications [133].

Recently, the first application of iPSC-PLTs in human circulation in vivo. It was conducted in the iPLAT1 study (jRCTa050190117) [134]. During the study, a clinical-grade iPSC-PLT production system was established. This study was designed as a single-center, open-label, uncontrolled dose-finding study of autologous iPSC-PLTs. The study involved only one patient, which may not reflect the impact of the produced cells on the population. The patient, a 50-year-old woman with aplastic anemia and allo-PTR immunization due to human platelet antigen (HPA)-1a antibodies, had not received transfusions previously, suggesting immunization occurred during pregnancy. Allo-PTR poses a significant clinical challenge as the PLT count does not rise even an hour after transfusion. Typically, such patients are treated with HLA class I or HPA-matched blood products. However, such products are not always available at blood centers, especially in emergencies. The same applies to autologous iPSC-PLTs produced for a specific patient. Their preparation time is 7–12 days [133]. During the iPLAT1 study, the patient received different doses of iPSC-PLTs. No clinically significant symptoms were observed after the administration of all doses. At the end of the one-year observation period following the last dose, the external Committee for Evaluation of Efficacy and Safety concluded that autologous iPSC-PLT administration was safe for the patient. Unfortunately, even after the transfusion of the maximum final dose, there was no significant increase in the patient’s PLT count. Moreover, shortly after the transfusion, a higher concentration of the d-dimer clotting marker was observed, which may suggest unexpected coagulation of iPSC-PLTs [134]. The research represents a notable advancement in the clinical utilization of iPSC-PLTs, facilitating the introduction of allogeneic alternatives and, leveraging their prospective advantages, augmenting the breadth of blood transfusion medicine.

### 4.2. Photobiomodulation

Studies have demonstrated that photobiomodulation using near-infrared (NIR) light, also known as low-level light therapy (LLLT), can significantly impact the quality and functionality of PLTs during storage and various medical procedures. Recently, Bontekoe et al. (2024) showed that the NIR treatment of PCs in PAS-E resulted in reduced PLT activation, evidenced by lower CD62P expression and reduced Annexin A5 binding. Additionally, there was decreased lactate production and higher pH levels, indicating a modulation of glycolysis, potentially through mitochondrial aerobic metabolism [135]. This aligns with the findings of Yang et al. (2016) and Zhang et al. (2016), who studied the effects of LLLT on immune thrombocytopenia in mice. They found that LLLT could alleviate immune thrombocytopenia by enhancing mitochondrial biogenesis in MKs and preserving mitochondrial function in PLTs, leading to increased PLT production and functionality [136,137].

Moreover, in vitro studies on whole blood performed by Walski et al. (2022) demonstrated that NIR PBM could reversibly inhibit PLT activation in a dose-dependent manner and reduce hemolysis, suggesting protective effects on both red blood cells and PLTs [138], which could be applied not only in storage but also in extracorporeal circulation systems [139,140,141].

In summary, NIR PBM exhibits significant potential in improving the quality and stability of stored PLTs through modulation of mitochondrial metabolism and protection against oxidative stress. These findings suggest promising applications for NIR and LLLT in enhancing PLT storage conditions and extending PLT shelf life in transfusion medicine.

### 4.3. Proteomic Analysis (Proteomics)—A Potential Biomarker for Platelets

Proteomic analysis refers to the systematic identification and quantification of the complete set of proteins (the proteome) of a biological system (cell, tissue, organ, biological fluid, or organism) at a specific point in time [142]. For proteomic analysis, the most often used technique is mass spectrometry [143].

Despite PLTs being unnucleated cells, they contain mRNA and possess all the necessary tools required for protein translation, inherited from their precursor cells, MKs. This characteristic has recently made PLTs the focus of proteomic studies [144].

In 2017, Rijkers et al. studied changes in PLTs during prolonged storage in standard conditions using label-free quantitative mass spectrometry and identified 21 proteins (out of 2501) that showed altered expression levels. Although the functionality of examined PLTs decreased, the changes in protein levels were relatively minor, suggesting that overall protein composition was only moderately affected. Additionally, the response to agonists was found to be reduced. During the study, no specific markers distinguishing ‘young’ or ‘old’ PLTs were identified. Only certain assumptions were made. For instance, it was observed that the expression of protein S100A9 rapidly declined on day 5 of storage, which may be indicative of young PLTs. Moreover, initial findings suggest that A2M, IGM, and GYG1 could potentially serve as biomarkers for ‘aged’ PLTs, but further investigation is required to validate this hypothesis [145].

Heililahong et al. conducted a research study where they performed a whole transcriptome analysis on stored PLTs on day 0, day 2, and day 4. They characterized mRNA, lncRNA, and circRNA profiles and discovered that differentially expressed RNA is closely related to PLT function, metabolism, DNA repair, cell cycle, and apoptosis. They identified certain candidate mRNAs as potential biomarkers for storage damage and observed that most of the pathways were related to DNA repair [146].

To analyze proteomic changes, various methods are employed. Examples include differential gel electrophoresis (DIGE), isotope-coded affinity tagging (ICAT), and isotope tagging for relative and absolute quantification (iTRAQ) [147]. In 2008, it was shown that utilizing different proteomic analysis techniques coupled with mass spectrometry yields varying results [61]. Only 5% of the findings were consistent across all methods used. Consequently, it is crucial to exercise caution when interpreting results obtained from a single-method analysis; nevertheless, proteomics, as an emerging and fascinating tool, holds promise for enhancing our understanding of the formation of PSL. It is also evident that the use of omics technologies can better match individual PCs to patients, potentially improving the efficiency and safety of transfusions. Through omics, also including metabolomics and transcriptomics, it is possible to gain a deeper understanding of the factors affecting the quality of stored PLTs and subsequently develop strategies to enhance their stability and extend their shelf life [146,148]. This may include modifications in storage procedures, the addition of appropriate protective substances, or genetic modifications.

### 4.4. miRNA Changes and PLTs Storage Conditions

miRNA is a small regulatory, single-stranded RNA (19–24 nucleotides) and was discovered in 1993. From that time on, more than 2500 have been defined [149]. They play a significant role in the post-transcriptional regulation of protein expression, affecting approximately one-third to half of human genes [150].

Recently, it was shown that miRNA plays an essential role in PLT regulatory functions in synthesizing proteins responsible for PLT activation and thrombus formation [151]. In 2009, Landry et al. found that P2Y_12_ mRNA is associated with Ago2·miRNA complexes in PLTs. They concluded that miRNAs might potentially exert coordinated and/or synergistic effects to regulate P2Y_12_ mRNA translation through its 3′untranslated regions (3′UTR). In such cases, miRNAs could play a crucial role in modulating PLT function [152].

A comprehensive study was conducted by Osman et al. in 2014 [113] to investigate the effects of three PI systems, namely INTERCEPT™, MIRASOL^®^, and gamma-irradiation, on the levels of miRNAs and mRNAs in PLTs stored in blood banks as well as their impact on PLT activation and function. The results revealed that 6 of the 11 studied miRNAs were already reduced by INTERCEPT™ on day 1. Both MIRASOL^®^ and INTERCEPT™ treatments resulted in a decrease in PLT count on day 1 of storage. Additionally, only the INTERCEPT™-treated PLTs exhibited a decrease in anti-apoptotic mRNAs within the first 24 h. The authors suggested that there is probably some specific mechanism related to miRNA changes in PI-treated PLTs. miRNA technology is relatively new compared to earlier designed PI systems, so it is advisable to stop the implementation of PI until its safety for miRNA is established.

Further investigations demonstrated that PLTs and PMPs possess distinct miRNA profiles. The authors suggest a specific miRNA loading into MPs during PLT activation [113,116]. Exposure of PLTs to INTERCEPT™ caused alterations in miRNA levels within PMPs, while MIRASOL^®^ treatment showed no significant effect on the miRNA profile in PLTs [112,113]. The authors suggested that changes in certain miRNA levels within PMPs derived from INTERCEPT™-exposed PLTs might contribute to the observed increased bleeding in some recipients.

Another group of researchers discovered that pathogen reduction technology may influence the miRNA profile of PMPs and induce overrepresentation of specific miRNAs [116]. They compared MIRASOL^®^ and INTERCEPT™ treatments, and the changes in miRNA were found in both cases. The conclusion from this study was to be careful with transfusing modified PLTs to patients, as the long-term consequences of using such altered materials remain unknown.

## 5. Big Data and Statistical Analysis in PLT Storage Management

Minimizing the wastage of blood and blood products is a crucial requirement all over the world. Two goals for a blood management system are the most important: ensuring product availability even in emergencies and reducing wastage resulting from product expiration. Managing PLT production is particularly challenging due to its short shelf life, leading to high rates of wastage and significant economic costs. Moreover, overproduction is common due to the inability of patients requiring PLT transfusion to wait for PC production.

The study conducted in 2016 by Sekhar et al. provides compelling evidence that effective management control can lead to improved concentrations of PC [129]. The authors presented data from a patient blood management program that involved the implementation of a service improvement initiative through the introduction of a ‘platelet coordinator’ role. The primary objective was to optimize the utilization of PLTs in a large, complex tertiary care hospital within the National Health Service setting. The study spanned three years and demonstrated a significant reduction in the supply and costs of PLTs. Specifically, between 2012/2013 and 2014/2015, there was a 21% decrease in both the number of PLT units supplied to the institution and the associated expenditure on PLTs. This reduction in PLT supply and costs was attributed to the implementation of enhanced strategies in waste management and stock control. These measures led to a more efficient utilization of the provided PLTs within the hospital. Despite an increase in overall hospital activity during the study period, the hospital managed to maintain stable PLT issue figures. Notably, the improvement in wastage control exceeded the improvements observed in the expenditure on PLTs, suggesting the successful optimization of PC usage [153].

Each country has its unique blood management system, which can vary in complexity depending on specific circumstances. Blood donation centers may support multiple hospitals or a dozen or more, making direct replication of solutions across countries unfeasible. In each case, it is necessary to conduct research and adapt the model to individual needs.

Nowadays, leveraging data analysis for optimizing PC production processes has become indispensable for the efficient utilization of healthcare resources. While the concept of modeling and estimating the demand for PCs is not new, with the first published studies dating back to the 1980s and 1990s [154,155], we now have access to greater computing power and artificial intelligence. Moreover, the ability to transfer and archive large amounts of data has become crucial for any modeling or machine learning endeavors.

In 2016, Pérez et al. published an article introducing the mathematical model they developed to enhance the management of PC production, aiming to make decision-making more rational and less empirical [125]. The data used for the model were derived from historical records of PCs produced, transfused, and discarded in the Basque Country in 2012. The model assumes a normal distribution of demand on each day of the week throughout the year, as previously observed in 2012. The authors presented an Excel spreadsheet where the estimation of the daily production of PCs was possible. They validated the model using real production data in 2013. The conclusion drawn was that the model served as a useful support tool during PC production, albeit with insufficient precision. Nevertheless, the results indicated a beneficial effect of modeling the data. Firstly, decisions could be made in a more rational manner rather than relying solely on empirical methods. Secondly, the model offered potential cost reductions of approximately ~0.5 million EUR annually. Lastly, the advantage of the model was that transfused units could be one day younger. The authors noted that for improved modeling precision, the implementation of a highly developed IT system is necessary. It would provide accurate information in real-time, thus enhancing the overall effectiveness of the model.

Guan et al. built and validated a statistical model for blood banking that enabled the prediction of PLT usage three days in advance. The data used for the study were obtained from the Stanford Blood Centre, which supplies all blood products for two associated hospitals: Stanford Health Care and Lucille Packard Children’s Hospital. The availability of data was facilitated by the implementation of a modern hospital electronic medical records (EMR) system. The research was based on finding the link between hospital-wide patient data and clinical transfusion decisions. The developed model considered data from 29 consecutive months and successfully reduced the expiration rate from 10.5 to 3.2%. It prohibits PLT shortages by reserving a minimum of 10 PC units on the shelf each day. The authors suggest that annually in this institution, the wastage of PCs could be reduced by 950 units without compromising patient care. If the same results were transferred nationally in the United States, the healthcare system could potentially save approximately 80 million USD [32].

**Figure 3:**
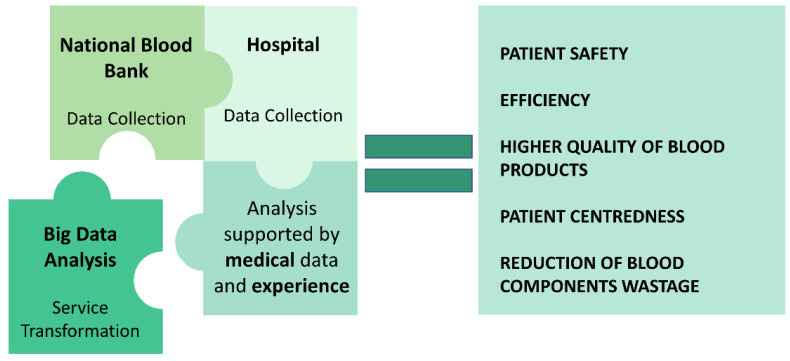
Harnessing the power of big data and statistical analysis to revolutionize platelet storage management: uncovering pathways to enhancement.

There is still limited research available in the field of big data and statistical analysis for PLT storage management with a solution ready to use. Conducting this type of study requires a substantial amount of data, and fortunately, it is becoming increasingly feasible to gather such data today. This area of research holds great promise and importance as it has the potential to significantly reduce blood and blood component wastage without relying on complex biological or additional techniques (Figure 3).

## 6. Conclusions

In recent years, much has been performed to better understand the effects of PLT storage. This brings with it improved transfusion safety and the possibility of better use of this valuable material. During the research, the database of clinical trials was checked. As of April 2024, the database contained records of 10 active clinical trials focused on PLT storage. These studies primarily revolve around two main objectives: cold storage (six trials) and cryopreservation (three trials). Additionally, one of the clinical trials focuses on investigating the impact of PLT storage on complement activation. Some specific topics of investigation include examining the impact of CD47 expression levels on PLTs stored at different temperatures [72], as well as studying CD62-P expression during storage and assessing the influence of preparation techniques [156]. Today, the world of science has access to tools and technologies that were not available a decade or so ago, offering new possibilities for the improvement of the PLT storage process. It would be worthwhile to determine whether these new findings can enhance the functionality of PLTs post-transfusion. The main goals for researchers in the next few years should be a better understanding of PSL, upgrading the banking methods of PLTs with the investigation of their in vivo quality, and employing big data modeling and artificial intelligence to estimate and predict PC production.

## Figures and Tables

**Table 2:** Limitations of current methods of PLTs storage in standard conditions and potential strategies for improvement.

Challenges of Standard Storage Conditions	State of Art	Solutions for Improvement
- Bacterial contamination [67,68,69,70]. - Constant agitation [64,65]. - Short time of storage (up to 5–7 days) [64,65,66]. - Strong PLT activation [8,16,17].	- PRT using UV light to prevent bacterial contamination [67,68,69,70]. - Gamma irradiation before transfusion [71,72]. - Constant agitation is necessary [65,66].	- Exploring novel PAS formulations to mitigate activation and bacterial contamination risks. - Investigating alternative materials for storage bags. - Evaluating different environmental conditions (such as air filtration).

**Table 3:** Limitations of current methods of PLTs cold storage and possible suggestions to improve.

Challenges of Cold Storage Conditions	State of Art	Solutions for Improvement
- PLT clearance caused by GPIbα-mediated signaling changes [78]. - PLT activation [65,83]. - Intracellular calcium release [65,83]. - α granules secretion [65,83]. - PLT morphology changes [65,83].	- Depletion of N-acetyl-2,3-dihydro-2-deoxyneuraminic acid and arachidonic acid prevents damage to cold-stored PLTs [78]. - Lower bacterial contamination [57]. - Good quality in vitro, not in vivo [28,84].	- Exploring the potential of reducing PLT activation through the utilization of novel PAS formulations and implementing lower storage temperatures. - Temporarily inhibiting the morphological transformation of PLTs. - Controlled calcium release by using new PAS components, for example Ligustrazine (Tetramethylpyrazine).

**Table 4:** Limitations of current methods of cryopreserved PLTs and suggestions for improvement.

Challenges of Cryopreservation	State of Art	Solutions for Improvement
- Length of PLT survival is shorter than 24 h [88,89]. - Almost no responsiveness to stimulation by agonists after storage [89,92]. - Changes in PLT phenotype [88]. - Microparticles formation [90].	- Storage with DMSO [88,90,92]. - Short-time efficiency is proven [92]. - Clinical trials are ongoing.	- Research for improved dilutions for storage at −80 °C. - Studies on preventing the decrease in glycoprotein levels. - Physicochemical research on inhibition of the PLTs activation.

**Table 5:** Limitations of platelet additive solutions and possible suggestions to improve.

Challenges of Storage in PAS	State of Art	Possible Solutions to Improve
- Studies mostly on in vitro quality of PAS [83]. - Citrate addition to PCs [26]. - Disturbed metabolism [26].	- Good quality in vitro [83]. - Lower risk of antibody incompatibility for the recipient (safer) [22,26].	- In vivo studies to verify how PAS influences PLTs. - Proteomic analysis to verify protein changes and long-term consequences.

## Data Availability

Not applicable.

## Abbreviations

ASCL	adipose-derived mesenchymal stromal/stem cell line
BC	buffy coat
CPP	cryopreserved platelets
DIGE	differential gel electrophoresis
DMSO	dimethyl sulfoxide
GP	glycoprotein
HPA	human platelet antigen
ICAT	isotope-coded affinity tagging
iPSC	induced pluripotent stem cells
iTRAQ	isotope tagging for relative and absolute quantification
LLLT	low-level light therapy
LP	lyophilized platelets
miRNA	microRNA
MK	megakaryocyte
PAMP	pathogen-associated molecular patterns
PAS	platelet additive solution
PC	platelet concentrate
PI	pathogen inactivation
PLT	platelet
PMPs	platelet microparticles
PPP	platelet-poor plasma
PRP	platelet-rich plasma
PSL	platelet storage lesion
TF	tissue factor
TRIM	transfusion-related immunomodulation
WHO	World Health Organization
